# Risk Assessment of Medical Study Procedures in the Documents Submitted to a Research Ethics Committee

**DOI:** 10.1177/1556264620903563

**Published:** 2020-02-08

**Authors:** Saara Happo, Tapani Keränen, Arja Halkoaho, Soili M. Lehto

**Affiliations:** 1 University of Eastern Finland, Kuopio, Finland; 2 Kanta-Häme Central Hospital, Hämeenlinna, Finland; 3 Tampere University of Applied Sciences, Finland; 4 University of Helsinki, Finland; 5 Helsinki University Hospital, Finland; 6 Kuopio University Hospital, Finland

**Keywords:** risk assessment, medical research ethics, medical study procedures, medical study protocol, participant information sheet

## Abstract

Several frameworks assist research ethics committees (RECs) in risk assessment of medical studies. However, little is known about how researchers describe risks of the procedures in study protocols and participant information sheets. We examined 349 study protocols and participant information sheets submitted to an REC and evaluated the risk assessments performed for 1,510 study procedures. Risks had been assessed for 399 (26%) procedures in study protocols and for 425 (28%) procedures in participant information sheets. Physical risks were assessed six times more frequently than psychological risks. Risks of medical procedures are not always detailed in study protocols or participant information sheets. Risk descriptions of procedures believed to be familiar to potential participants may be omitted.

## Introduction

Medical research may expose participants to risks. However, as stated in the Declaration of Helsinki (World Medical Association, 2013) and other international guidelines such as ICH harmonized tripartite guideline: Guideline for good clinical practice (ICH-GCP, 1996), all the risks involved in any medical study need to be both evaluated and justified before study initiation. Furthermore, no physician should undertake any research in which the risks have not been thoroughly assessed (World Medical Association, 2013). Both researchers and research ethics committees (RECs) need to evaluate the risks to which study participants will be exposed. REC members conduct the risk assessment according to the information they are able to extract from the study protocol provided to them by the researchers. Furthermore, all risks should be explained to all potential participants before asking them to provide informed consent (World Medical Association, 2013).

The possibility of risk varies according to the chosen study procedures. Study procedures used in medical research can be divided into therapeutic interventions and into nontherapeutic study procedures intended for data collection (Weijer & Miller, 2004; Wendler & Miller, 2007). In cases of therapeutic interventions and nontherapeutic experimental interventions, the risks of study procedures may not always be known. Nontherapeutic study procedures necessary for data collection usually involve common procedures in health care, which means that their possible risks, likelihood, and intensity of possible harm, are more predictable. Possible risks can be divided into physical risks and psychological risks and further into financial, legal, and experiential risks (Resnik, 2017).

Several methods and frameworks have been developed to assist RECs to conduct their risk assessments (Bernabe et al., 2012; Miller, 2012; Rajczi, 2004; Rid et al., 2010, 2014; Rid & Wendler, 2011a; Wendler & Miller, 2007). In all cases, researchers are assumed to have carefully detailed all possible risks in the study protocols and participant information sheets that are submitted to REC. However, researcher-provided descriptions of study risks may remain unsatisfactory due to several reasons, such as lack of experience or inadequate training in risk evaluation. If a study protocol does not provide sufficient information to the REC about the risks inherent in the proposed study, the risk assessment may be conducted subjectively or have to be based on intuition (Resnik, 2017). Moreover, incomplete risk descriptions may also lead to a delayed handling of the application because the REC may need to ask for clarifications regarding the possible risks (Adams et al., 2013; Resnik, 2017; Happo et al., 2017).

Little research has been conducted on the adequacy of risk assessment for study procedures included in clinical study protocols, or on the description of risks in participant information sheets, submitted for REC assessment. Data from approved clinical study protocols and participant information sheets have been extracted to record possible complications associated with biopsies taken for research purposes (Overman et al., 2013). It was observed that the risk descriptions of research biopsies were not systematically described in either the study protocols or the participant information sheets. Furthermore, investigator’s brochures have been examined to find out whether they permit an appropriate assessment of the risk to the participants (Wieschowski et al., 2018). That report concluded that the investigator’s brochures did not provide sufficient information about preclinical efficacy to conduct an appropriate risk–benefit assessment. To the best of our knowledge, risk assessment studies that simultaneously examine multiple study procedures common in health care research have not been conducted before.

Here, we evaluated a sample of 1,510 study procedures commonly conducted in research in health care and medical practice. We sought to examine whether the possible risks of study procedures had been described in (a) study protocols and (b) participant information sheets.

## Method

### Study Sample

Data on the procedures examined were extracted from documents corresponding to 349 clinical study protocols derived from the records of the REC of North Savo Hospital District, Finland, from January 1, 2009, to December 31, 2013. Altogether, our data consisted of 1,510 study procedures corresponding to common procedures in health care and medical research. The data have been described in detail elsewhere (Happo et al., 2017).

In brief, the utilized study protocols contained both a separate principal investigator’s ethics statement, in this study included in the protocol, and a participant information sheet. The focus of this study was on the original risk assessments of the study procedures, and therefore, we surveyed the documents in the format in which they had been initially submitted for REC assessment. Amendments to study protocols were not assessed in our study. Thus, any REC-requested amendments to study protocols were excluded from our study. Therefore, these data provide a more direct view on the researchers’ perspectives than the amended documents. The final accepted documents would have been corrected in line with the REC’s amendment requests, and thus no longer directly reflect researchers’ views on how risks should be presented.

### Identification and Classification of Study Procedures

**Figure 1.**
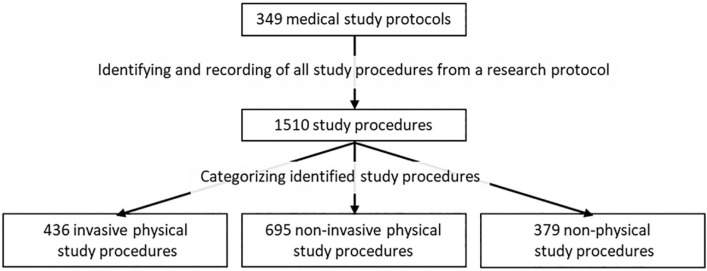
Identifying and categorizing of study procedures.

**Figure 2.**
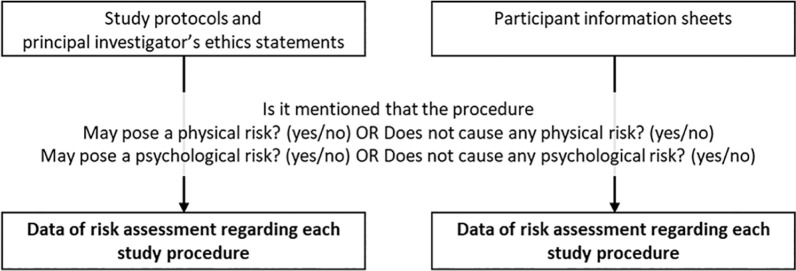
Data collection of risk assessment regarding study procedures.

The data collection procedures are described in Figures 1 and 2. First, we identified each study procedure to be performed on the participants as recorded in the study protocol (Figure 1). In the REC’s assessment, all study procedures are expected to be described in a study protocol to enable the committee to assess the study’s scientific quality. It is assumed that the same study procedures are described in the participant information sheet attached to the protocol. Second, we classified the identified study procedures into invasive physical procedures, noninvasive physical procedures, and nonphysical procedures according to the risk they may have posed to the participant. The coding procedures regarding the placement of different types of procedures into the specific procedure categories were agreed on a priori basis as a consensus by the author team; subsequently, the first author then conducted the practical work following the predefined consensus manual. In case of any uncertainties regarding the procedure, the main author consulted the rest of the project team to ensure that the agreed procedure was accurately and consistently followed.

We defined an invasive physical study procedure as *a medical study procedure that invades the body*, *by cutting or puncturing the skin*, *or by inserting instruments into the body*; *introduction of investigational drugs or nutritional agents*; *or exposing the body to medical radiation*. These study procedures (e.g., study drug administration, nutritional experiments, blood sampling, and X-ray imaging) make a participant more susceptible to some risk, that is, not only to physical harm, but also to psychological effects, than nonparticipation in the study.

Noninvasive physical study procedures were defined as *procedures that target the participant’s physique or physical health but do not involve any tools or introduction of agents that break the skin or physically enter the body* (e.g., prescription of physical exercises to be done at home, weighing and other body measurements, magnetic resonance imaging, and urine sampling). The noninvasive physical procedures did not to expose participants to any physical risk greater than everyday life but may have potentially posed some psychological risk.

Nonphysical study procedures were defined as *procedures that involve no study-related physical procedures on the participant*, *and they can be carried out without touching or causing movement or prescribing exercise for the participant* (e.g., questionnaires, interviews, and psychological interventions). Therefore, they posed no physical risk to the participant but may have potentially posed a psychological risk.

All of the above study procedures were divided further into therapeutic interventions as follows (a) experimental interventions, (b) medical devices under investigation, and (c) study drug administrations (Supplemental Table 1), and into nontherapeutic data collection procedures including (a) taking biological samples, (b) imaging, and (c) measurements and examinations (Supplemental Table 2). The categorization and the numbers of identified study procedures are presented in Supplemental Tables 1 and 2.

To study whether likely familiarity with a procedure to a potential participant would have an impact on the risk description or risk assessment in the study documents, study procedures were categorized to (a) procedures that were likely to be familiar to an average participant and to (b) procedures that were are not likely to be familiar to an average participant. Procedures were considered to be likely familiar to a potential participant if (a) a procedure was common in everyday life (such as filling a questionnaire or weighing and other body measurements) or (b) a procedure was unambiguous to understand to an average participant (such as taking a blood or urine sample or monitoring basic body functions). The categorization and the number of each identified study procedure are presented in Supplemental Table 3.

### Data Collection of Risk Assessment Regarding Study Procedures

After the identification and classification of the study procedures, we examined the study-related documents (i.e., study protocols with principal investigators’ ethics statements and participant information sheets) to evaluate the principal investigators’ description on risks or statements regarding the lack of risks in the proposed study procedures (Figure 2). In this study, based on the content of the reviewed protocols, we made a distinction between those where a general assessment of risk (including lack of risk) had been made and those where a risk had been specifically described. A risk description means that a study procedure’s possible risk or risks had been written down in a study protocol or participant information sheet (yes/no). For example, sentences such as “A blood draw exposes a participant to a minor risk such as pain or hematoma” or “You may feel minor pain when the blood sample is taken.” represented a risk description. Risks were considered as having been assessed (yes/no) if there was (a) a description of a possible harm that a procedure might pose to the participant, as described above (e.g., when there is some description of the risks, that is, they are considered to have been assessed), or (b) there was a statement that a procedure would not cause any harm to a participant (i.e., in these cases, there had been a risk assessment but no risk description). For example, sentences such as “Taking a swap sample does not involve any risk.” or “Taking a swap sample will not cause you any pain.” means that the risk assessment was conducted. We concluded that the risk assessment was lacking when (a) there were no descriptions of a procedure’s potential risk (i.e., there was no risk description and no risk assessment) or (b) there was no mention that a procedure would not cause any harm to a participant (i.e., there was no risk description and no risk assessment). In this study, we did not evaluate the accuracy or sufficiency of the risk descriptions.

The risk descriptions were further divided into physical and/or psychological risks based on the principal investigator’s descriptions of the estimated risk. The principal investigator’s description of estimated risks associated with one procedure could include physical risks, or psychological risks, or both types of risks.

Physical risk was defined as a potential harm that causes an immediate physical response such as pain, feeling of pressure, or nausea, or a delayed response such as increased risk of infection or cancer. Psychological risk was determined to be immediate psychological harm (e.g., feeling uncomfortable or fearful) or potential long-term harm (e.g., depression or an increased risk of suicide). Risks possibly caused by participation itself, such as social consequences, loss of time, possible travel costs, or risks attributable to personal data security were not examined in this study.

### Statistical Analyses

Statistical differences between different study procedure categories among invasive physical study procedures and among noninvasive physical study procedures were tested with chi-square and Fisher’s exact tests. To examine whether a specific procedure category would be handled differently than the others, the number of risks descriptions or risk assessments in each study procedure category was compared with the mean number of risk descriptions or risk assessments in other procedure categories within (a) invasive physical study procedures and (b) noninvasive physical study procedures. Two-tailed *p*-values below .05 were considered statistically significant. The software R (version 3.5.1, from the R Foundation for Statistical Computing, 2018) was used for statistical analysis. Data are shown as frequencies and percentages.

## Results

### Risk Assessments of Study Procedures Presented in Study Protocols

The risk assessments of the procedures recorded in study protocols are presented in Table 1. In total, risks of only 399 (26%) study procedures were assessed in the study protocols. Physical risks of the procedures were described in the study protocols on average six times more frequently than psychological risks. Furthermore, in 66 cases (4.4%), although a study procedure’s risks had been described in the study protocol, there was no mention of the corresponding procedure’s risks in the participant information sheet.

**Table 1.** Frequency of Risk Descriptions and Risk Assessment in Study Protocols, *n* (%), With Statistically Significant *p*-Values for Each Column of Invasive Physical Procedures and Each Column of Noninvasive Physical Procedures Derived From Chi-square Tests and Fisher’s Exact Tests.

Type of a procedure	*n*	All risks		Physical risks		Psychological risks		Risks described only in the study protocol
		Risks described^a^	Risks assessed^b^	Risks described^a^	Risks assessed^b^	Risks described^a^	Risks assessed^b^	
Invasive physical interventions								
Experimental interventions	59	22 (37)	33 (56)	21 (36)	33 (56)	1 (1.7)	2 (3.4)	6 (10)
Study drug administration	73	32 (44)	43 (59)	31 (42)	42 (58)	9 (12)*	13 (18)**	0 (0)**
Medical device under investigation	17	6 (35)	9 (53)	6 (35)	9 (53)	0 (0)	0 (0)	1 (5.9)
Taking biological samples	199	73 (37)	99 (50)	69 (35)	94 (47)	15 (7.5)	16 (8.0)	21 (11)
Imaging	49	26 (53)	37 (76)**	26 (53)*	35 (71)**	2 (4.1)	2 (4.1)	6 (12)
Measurements and examinations	39	6 (15)**	12 (31)**	6 (15)**	11 (28)**	2 (5.1)	3 (7.7)	2 (5.1)
	436	164 (38)	233 (53)	159 (36)	224 (51)	29 (6.7)	36 (8.3)	36 (8.3)
Noninvasive physical procedures								
Experimental interventions	13	2 (15)	5 (38)	2 (15)	5 (38)^+^	1 (7.7)	1 (7.7)	0 (0)
Medical device under investigation	27	1 (3.7)	15 (56)^++^	0 (0)	15 (56)^++^	1 (3.7)	1 (3.7)	1 (3.7)
Taking biological samples	128	8 (6.3)	21 (16)	1 (0.8)	15 (12)	6 (4.7)	7 (5.5)	5 (3.9)
Imaging	63	7 (11)	29 (46)^++^	1 (1.6)	26 (41)^++^	7 (11)^++^	9 (14)^++^	3 (4.8)
Measurements and examinations	464	30 (6.5)	55 (12)^++^	21 (4.5)	44 (9.5)^++^	10 (2.2)^++^	13 (2.8)^++^	13 (2.8)
	695	48 (6.9)	125 (18)	25 (3.6)	105 (15)	25 (3.6)	31 (4.5)	22 (3.2)
Nonphysical procedures								
Experimental interventions	12	1 (8.3)	1 (8.3)	0 (0)	0 (0)	1 (8.3)	1 (8.3)	1 (8.3)
Measurements and examinations	367	15 (4.1)	40 (11)	0 (0)	21 (5.7)	13 (3.5)	30 (8.2)	7 (1.9)
	379	16 (4.2)	41 (11)	0 (0)	21 (5.5)	14 (3.7)	31 (8.2)	8 (2.1)
TOTAL	1,510	229 (15)	399 (26)	184 (12)	350 (23)	68 (4.5)	98 (6.5)	66 (4.4)

a Risks are described in the study protocol. ^b^Risks or lack of risks of the procedure are mentioned in the study protocol.

Statistical differences in the number of risk descriptions or risk assessments in the marked procedure group compared with the mean of other procedure groups in the same column related to invasive physical procedures, *p-value < .05; **p-value < .01.

Statistical differences in the number of risk descriptions or risk assessments in the marked procedure group compared with the mean of other procedure groups in the same column related to noninvasive physical procedure groups, ^+^*p*-value < .05; ^++^*p*-value < .01.

The number of risk descriptions or risk assessments in each study procedure category was compared with the mean number of risk descriptions or risk assessments in the other procedure categories.

In the category of invasive physical study procedures, risks had been assessed in 233 (53%) procedures and risks were described for 164 (38%) of the invasive physical study procedures. It was uncommon that there had been any mention of risk assessments for noninvasive physical study procedures and nonphysical procedures in study protocols, noninvasive physical study procedures: *n* = 125 (18%); nonphysical study procedures: *n* = 41 (11%).

It was observed that the risks related to medical imaging procedures were assessed more frequently than the risks associated with other study procedure categories. We observed the same phenomenon in both the category of invasive imaging procedures (risks assessed for 37 imaging procedures; 76%), such as X-ray imaging, and the category of noninvasive imaging procedures (risks assessed for 29 imaging procedures; 46%), such as ultrasound and magnetic resonance imaging (MRI). In contrast, the risks of examinations and measurements (e.g., physician examination, cardiac stress test, or electrocardiography) were assessed less frequently (risks assessed for 12 examinations and measurements; 31%) than risks of other study procedure categories such as the collection of biological samples (risks assessed for 99 sample collection; 50%) or the exposure to experimental interventions (such as nutritional interventions or physiological exercise, risks assessed for 33 interventions; 56%). Among noninvasive physical procedures, risks related to the utilization of medical devices under investigation (risks assessed for 15 medical devices; 56%) were assessed more frequently than in the other study procedure groups.

### Risk Assessments of Study Procedures Presented in Participant Information Sheets

The risks involved in the study procedures written down to the participants in the participant information sheets are presented in Table 2. In total, a description of the risks or lack of risks had been recorded in participant information sheets for 427 (28%) study procedures, with 301 (20%) of the procedures actually stating that the procedure did pose a possible risk to a participant. Physical risks were described for 263 (17%) study procedures and psychological risks described for 134 (8.9%) study procedures. In 136 (9.0%) cases, the risks of a study procedure had been described to the participant in the information sheet, but not described in the study protocol. This phenomenon was more frequent in the case of invasive physical study procedures (risks for 88 invasive physical study procedures were described in the participant information sheet but not in the study protocol; 20%). It especially occurred when the study involved drug administration (risks for 29 study drug administrations were described in the participant information sheet but not in the study protocol; 40%).

**Table 2.** Frequency of Risk Descriptions and Risk Assessment in Participant Information Sheets, *n* (%), With Statistically Significant *p*-Values for Each Column of Invasive Physical Procedures and Each Column of Noninvasive Physical Procedures Derived From Chi-square Tests and Fisher’s Exact Tests.

Type of a procedure	*n*	All risks		Physical risks		Psychological risks		Risks described only in the participant information sheet
		Risks described^a^	Risks assessed^b^	Risks described^a^	Risks assessed^b^	Risks described^a^	Risks assessed^b^	
Invasive physical interventions								
Experimental interventions	59	20 (34)*	36 (61)	19 (32)*	35 (59)	6 (10)*	6 (10)*	4 (6.8)*
Study drug administration	73	61 (84)**	61 (84)**	61 (84)**	61 (84)**	34 (47)**	34 (47)**	29 (40)**
Medical device under investigation	17	7 (41)	9 (53)	7 (41)	9 (53)	0 (0)*	0 (0)*	2 (12)
Taking biological samples	199	93 (47)	110 (55)	88 (44)	103 (52)*	43 (22)	43 (22)	41 (21)
Imaging	49	27 (55)	32 (65)	25 (51)	30 (61)	4 (8.1)*	4 (8.1)*	6 (12)
Measurements and examinations	39	10 (26)**	12 (31)**	10 (26)**	12 (31)**	3 (7.7)*	3 (7.7)*	6 (15)
	436	218 (50)	260 (60)	210 (48)	250 (57)	90 (21)	90 (21)	88 (20)
Noninvasive physical procedures								
Experimental interventions	13	2 (15)	2 (15)	2 (15)	2 (15)	1 (7.7)	1 (7.7)	0 (0)
Medical device under investigation	27	1 (12)	12 (44)^++^	1 (12)	12 (44)^++^	0 (0)	0 (0)	1 (3.7)
Taking biological samples	128	9 (7.0)	24 (19)	2 (1.6)^++^	18 (14)	9 (7.0)	11 (8.6)^+^	6 (4.7)
Imaging	63	10 (16)	43 (68)^++^	2 (3.2)	40 (63)^++^	9 (14)^++^	10 (16)^++^	6 (10)
Measurements and examinations	464	44 (9)	63 (14)^++^	46 (10)^++^	56 (12)^++^	8 (1.7)^++^	10 (2.2)^++^	28 (6.0)
	695	66 (9.5)	144 (21)	53 (7.6)	128 (18)	27 (3.9)	32 (4.6)	41 (5.9)
Nonphysical procedures								
Experimental interventions	12	0 (0)	1 (8.3)	0 (0)	0 (0)	0 (0)	0 (0)	0 (0)
Measurements and examinations	367	17 (4.6)	22 (6.0)	0 (0)	4 (1.1)	17 (4.6)	20 (5.4)	7 (1.9)
	379	17 (4.5)	23 (6.0)	0 (0)	4 (1.1)	17 (4.5)	20 (5.3)	7 (1.8)
TOTAL	1,510	301 (20)	427 (28)	263 (17)	382 (25)	13 (8.9)	142 (9.4)	136 (9.0)

a Risks are described in the participant information sheet. ^b^Risks or lack of risks of the procedure are mentioned in the participant information sheet.

Statistical differences in the number of risk descriptions or risk assessments in the marked procedure group compared with the mean of other procedure groups in the same column related to invasive physical procedures, *p-value < .05; **p-value < .01.

Statistical differences in the number of risk descriptions or risk assessments in the marked procedure group compared with the mean of other procedure groups in the same column related to noninvasive physical procedure groups, ^+^*p*-value < .05; ^++^ *p*-value < .01.

The number of risk descriptions or risk assessments in each study procedure category was compared with the mean number of risk descriptions or risk assessments in the other procedure categories.

A total of 218 (50%) of invasive physical study procedures were described in participant information sheets to pose risks to the participants. The corresponding figures for noninvasive physical study procedures were 66 (9.5%) and for nonphysical study procedures 17 (4.5%).

When focusing on the invasive physical study procedures, it seemed that the risks of a study drug administration had been described most frequently to the participants (risks described for 61 study drug administrations; 84%). In contrast, the risks of measurements and examinations were more rarely assessed (risks assessed for 12 procedures; 31%) and described (risks described for 10 procedures; 26%) than in other invasive physical study procedure categories. The potential risks of invasive experimental interventions, such as nutritional interventions or surgical experiments, had been commonly assessed in the participant information sheets (risks assessed for 36 interventions; 61%), but both physical and psychological risks were described to be present less frequently (physical risks described for 19 procedures; 32%, psychological risks described for six procedures; 10%) in comparison with the other invasive physical study procedure categories.

With respect to noninvasive physical study procedures, the risks associated with medical devices under investigation and imaging had been more frequently assessed (risks assessed for 12 medical devices; 44%, and for 43 imaging procedures; 68%) than other study procedures, but they were not described to pose risks more frequently (risks described for one medical device; 12%, and for 10 imaging procedures; 16%) than procedures in the other noninvasive physical study procedure categories.

### Study Procedures’ Familiarity to a Potential Participant

Table 3 shows the relationship between “likely familiarity” of a procedure and the rates at which risks for this procedure were either assessed or described in the protocol. In both study protocols and participant information sheets, risks of procedures which were thought to be unfamiliar to a potential participant had been presented on average three times more often.

**Table 3.** Frequency of Risk Descriptions and Risk Assessment of Study Procedures Classified According to Likely Familiarity to an Average Participant in Study Protocols and Participant Information Sheets, *n* (%).

Type of a procedure	*n*	Study protocols		Participant information sheets	
		Risks described^a^	Risks assessed^b^	Risks described^a^	Risks assessed^b^
Study procedures likely to be familiar to an average participant	979	92 (9.3)	161 (16)	113 (12)	156 (16)
Study procedures likely to be nonfamiliar to an average participant	531	137 (26)	238 (45)	188 (35)	271 (51)
TOTAL	1510	228 (15)	399 (26)	184 (12)	350 (23)

a Risks are described in the study protocol or in the participant information sheet. ^b^Risks or lack of risks of the procedure are mentioned in the study protocol or in the participant information sheet.

## Discussion

This study has surveyed both medical study protocols and participant information sheets submitted for REC assessment over a time span of 5 years. As the documents represented those submitted for the initial REC assessment, the investigators had received no systematic guidance on conducting a risk assessment. Therefore, the protocols and their attachments represented the investigators’ views about how they had assessed and then described the risks. Our main finding was that even though there were procedures that did pose risks to the participants, these potential risks were not always described in the study documents.

In the ideal scenario, the risk assessment would comprise descriptions of likelihood and the intensity of possible harm of each procedure to be conducted (Rid et al., 2010). In particular, invasive data collection procedures may expose participants to risks without any prospects of health benefits, and therefore the risks need to be scientifically justified (Kimmelman et al., 2017), for example, to hold the possibility of benefiting society as a whole (Rid & Wendler, 2011a; Wendler & Miller, 2007; World Medical Association, 2013). In the case of experimental interventions, the risks may even be somewhat unknown.

In our study, risks had been assessed for 233 (53%) of invasive study procedures described in the study protocols, and specifically described in a smaller percentage (38%). Nevertheless, even though these values appear very low, they are in line with one previous study (Overman et al., 2013) that highlighted the need for a more comprehensive presentation of risks and benefits for the study protocols and the participant information sheets. The final number of procedure risk descriptions is likely to be higher. The evaluated documents in our study represent the original, nonamended batch of documentation submitted to an REC.

One of an REC’s main tasks is to estimate a risk–benefit ratio of the proposed study (World Medical Association, 2013). There are several frameworks available for procedure-level risk assessments (Rid et al., 2010, 2014; Rid & Wendler, 2011a; Wendler & Miller, 2007). In a previous report (Van Luijn et al., 2002), REC members considered that they did not obtain adequate information about a study’s risks to allow them to decide whether the risk–benefit ratio would be favorable. The results of this study support this finding: REC members may not be able to make an appropriate risk benefit assessment without time-consuming amendment requests, if the risk descriptions of procedures remain less than comprehensive. According to another study (Wao et al., 2014), if a study’s risks are assessed to be low, this will exert a positive influence on its acceptance by the REC. If a study protocol contains a low number of risk descriptions in comparison to the actual, potential risks, it may be accepted with insufficient attention being paid by the REC to participant safety.

The prerequisite for an informed consent is that potential participants should be supplied with a written explanation of risks (ICH-GCP, 1996; World Medical Association, 2013). We found out that even when using invasive study procedures, the risks associated with invasive study procedures had been assessed for only 260 (60%) procedures in participant information sheets. Furthermore, only every second of invasive procedures had been stated to pose any risks to the participants in the information sheets.

RECs’ task is to ensure that the risk benefit ratio of a study is acceptable. However, the final decision regarding study participation and accepting the risks is left to the discretion of each potential participant (Shaw, 2014; Van Luijn et al., 2002). Therefore, REC members need to ensure that the potential risks of procedures have been appropriately described in participant information sheets, even when they might be minor, uncertain, or even controversial (DeMarco et al., 2014). The risks should also be described in a manner that is easily understandable for each potential participant. In particular, some sections of participant information sheets may be rather difficult to fully comprehend. In a previous report, participants were less able to understand the risks and insurance policy–related issues of clinical trials than the other details of the study protocol (Hereu et al., 2010).

As expected, the risks of invasive physical study procedures were more frequently assessed in both study protocols and participant information sheets than the risks of noninvasive physical procedures or nonphysical study procedures. Our findings are in accordance with previous observations indicating that potential risks associated with low-risk study procedures are reported more rarely than the risks of high-risk study procedures (Rid & Wendler, 2011b). Overall, psychological risks were less likely to have been assessed and recorded than physical risks in both study protocols and participant information sheets. In the participant information sheets, psychological risks had been assessed for 90 (21%) invasive physical study procedures, but only for 32 (4.6%) noninvasive physical study procedures, and for 20 (5.3%) nonphysical study procedures. In the study protocols, the figures were even lower for physical study procedures. These values may indicate that researchers are more aware of the physical risks than their psychological counterparts. For many study procedures, there may not be as much scientific evidence on a procedure’s psychological risks as there are for its physical risks. Therefore, researchers may also consider psychological risks as clearly less important than physical risks. Nevertheless, in some settings, psychological risks may hold a high level of relevance for a participant. For example, noninvasive imaging, such as magnetic resonance imaging, is known to cause psychological risks such as feelings of claustrophobia (Marshall et al., 2007; Versluis et al., 2013). Furthermore, obtaining invasive research samples, such as performing a lumbar puncture, may expose participants to both physical and psychological risks (Peskind et al., 2005). In addition, research on sensitive topics such as experienced sexual violence may be associated with certain psychological risks such as distress or traumatic memories in the participants (Decker et al., 2011).

Guidelines such as the Declaration of Helsinki (World Medical Association, 2013) and ICH-GCP (1996) emphasize that researchers always need to include descriptions of potential risks in the study documentation. Nevertheless, in our study, the frequency of presenting risk assessments for study procedures remained low both in study protocols (risks assessed in only 399 [26%] study procedures) and in participant information sheets (risks assessed in only 425 [28%] study procedures). Our analyses focusing on the familiarity of study procedures suggest that there may well be pragmatic reasons behind these apparently unsatisfactory values. The most common invasive physical study procedure in our study was blood sampling, used 147 times (9.7% of all study procedures and 34% of invasive physical study procedures), and the most common noninvasive physical study procedure was weighing or some other similar body measurements, used 111 times (7.4% of all study procedures and 16% of noninvasive physical study procedures). In addition, questionnaires had been used 169 times as a research procedure, representing 11% of all study procedures and 45% of the nonphysical study procedures. All these procedures are very common in both clinical practice and health care research, and consequently perhaps expected to be familiar to potential participants. In addition, in a total of 281 (79%) studies, the participant group had been patients (Happo et al., 2017). It can be assumed that procedures conducted for research purposes may also have been used in the participants’ standard care. Researchers may therefore consider that the study procedures would be more familiar to these kinds of subjects than to other participants. Such underlying expectations may guide the manner in which the researchers present risks related to these very commonly used study procedures.

Some limitations need to be taken into consideration while interpreting our findings. We were able to only gain access to a 5-year period of all study documentations from one REC; if it had been possible to access the documentation of several RECs, this would have improved the generalizability of our data. However, our data of 1,510 study procedures represented well the study procedures most typical for medical and health care research. In addition, a total of 72 (21%) of the studies from which the procedures were derived represented international multicenter studies, and 54 (15%) were national multicenter studies. Therefore, the derived study material represents data corresponding to an area that is larger than the catchment area of the REC that acted as a data source (i.e., Central-Eastern Finland; Happo et al., 2017). In addition, as our sample dates to years 2009 to 2013, it is possible that the style of risk assessment of study procedures has changed. However, the written instructions on risk assessment, provided for researchers by the target REC online as well in the Finnish Medical Research Act (1999), have remained unchanged since 2009. Therefore, we interpret that our observations reflect current researcher practices.

In 29 (40%) of all the clinical drug trials examined, one or more possible risks of an investigational drug had been listed only in the participant information sheet, but not in the clinical drug trial protocol. However, our study materials did not include investigator’s brochures that contain preclinical and clinical evidence on safety and tolerability of an investigational drug. Therefore, it may be that some of the clinical drug trials had provided risk descriptions only in the investigator’s brochures. Nevertheless, the ICH-GCP guideline requires “*a summary of the known and potential risks and benefits, if any, to human subjects*” of a clinical drug trial to be included in a study protocol. Therefore, it is highly likely that all of the risks possibly described in the investigator’s brochures have also been described in the study protocols or, as our data shows, even more likely in a participant information sheets.

In conclusion, we observed that risks of only slightly more than one out of every four of the all medical research procedures had been described in study protocols or detailed in participant information sheets at the time when the protocol had been first submitted to the REC. These findings may represent a conflict between the recommended best practice and researchers’ everyday practices: the researchers may leave out risk descriptions of procedures that they assume should be familiar to potential participants. Our findings also imply that researchers communicate risks differently to participants in the information sheets and to the academic community (i.e., here, RECs) in study protocols, or that they interpret research ethics guidelines in a manner that suggests that it is sufficient to only describe procedure risks in one document type submitted to an REC.

## Best Practices

A well-rounded risk assessment and a clear presentation of possible risks to potential participants are essential in medical research. Our present study suggests that risk assessment had been conducted only for approximately one quarter of the used study procedures. The study protocols from which we assessed the data on risk assessment of medical procedures represented a mix of local, national, and international studies. However, somewhat less than two thirds of the studies had been conducted locally in the data provider REC’s catchment area, which may limit the generalizability of our findings. We recommend that both researchers and REC members should ensure that physical and psychological risks are appropriately assessed in study documents to ensure that all possible risks are considered in the risk–benefit assessment at the level of the whole study. Furthermore, all risks described in a study protocol should be recorded in the information sheet provided to participants, as they are important in allowing the individual to decide whether or not he or she will provide consent to participate in a study. Further discussion within the medical research ethics community is also warranted regarding whether the familiarity of certain study procedures to the general public affects how researchers and RECs deem it necessary to include risk descriptions of these procedures in study protocols and participant information sheets.

## Research Agenda

Inadequately described risks of study procedures have been reported to be one of the most common reasons why RECs request clarifications or demand amendments to research protocols and participant information sheets (Adams et al., 2013; Dal-Re et al., 2004; Lopez-Parra et al., 2012; Happo et al., 2017), and our present findings are in line with these observations. However, there is still limited knowledge as to why researchers appear to focus on the presentation of certain potential study procedures risks more than others. To clarify this issue, researchers’ views on risk assessment of common medical study procedures should be examined.

## Educational Implications

Risk assessment of medical procedures in the study protocols and participant information sheets submitted for initial REC assessment appears to be rather nonsystematic. RECs may want to routinely provide researchers with more detailed instructions on how to assess the risks associated with certain types of study procedures in both study protocols and participant information sheets; such practices would likely facilitate the REC’s own evaluation processes. Furthermore, it has been reported that enhanced support to researchers is one way to improve the acceptance rate from RECs (Sonne et al., 2018). Further education on risk assessment of medical study procedures is recommended, in particular for early stage researchers.

## Supplemental Material

SupplementaryTable1 – Supplemental material for Risk Assessment of Medical Study Procedures in the Documents Submitted to a Research Ethics Committee Click here for additional data file. Supplemental material, SupplementaryTable1 for Risk Assessment of Medical Study Procedures in the Documents Submitted to a Research Ethics Committee by Saara Happo, Tapani Keränen, Arja Halkoaho and Soili M. Lehto in Journal of Empirical Research on Human Research Ethics

SupplementaryTable2 – Supplemental material for Risk Assessment of Medical Study Procedures in the Documents Submitted to a Research Ethics Committee Click here for additional data file. Supplemental material, SupplementaryTable2 for Risk Assessment of Medical Study Procedures in the Documents Submitted to a Research Ethics Committee by Saara Happo, Tapani Keränen, Arja Halkoaho and Soili M. Lehto in Journal of Empirical Research on Human Research Ethics

SupplementaryTable3 – Supplemental material for Risk Assessment of Medical Study Procedures in the Documents Submitted to a Research Ethics Committee Click here for additional data file. Supplemental material, SupplementaryTable3 for Risk Assessment of Medical Study Procedures in the Documents Submitted to a Research Ethics Committee by Saara Happo, Tapani Keränen, Arja Halkoaho and Soili M. Lehto in Journal of Empirical Research on Human Research Ethics
