# Supplementary material for: Risk Assessment of Medical Study Procedures in the Documents Submitted to a Research Ethics Committee
Source: J Empir Res Hum Res Ethics. 2020 Feb 8;15(5):396–406. doi: 10.1177/1556264620903563 (PMC7604935; doi:10.1177/1556264620903563)
Supplement: SupplementaryTable1 – Supplemental material for Risk Assessment of Medical Study Procedures in the Documents Submitted to a Research Ethics Committee [file SupplementaryTable1.pdf]

**Supplementary Table 1: Therapeutic interventions (n=201; 13%)**		
**Invasive physical procedures (n=149; 10%)**		
Experimental interventions (n=59; 3.9%)	Medical device under investigation (n=17; 1.1%)	Study drug administration (n=73; 4.8%)
Nutritional interventions (n=31; 2.1%)	Therapeutic device (n=10; 0.7%)	Phase I (n=3; 0.2%)
Transcranial magnetic or electric stimulation (n=10; 0.7%)	Diagnostic device (n=7; 0.5%)	Phase II (n=9; 0.6%)
Surgical experiment (n=8; 0.5%)		Phase III (n=37; 2.5%)
Halotherapy (n=3; 0.2%)		Phase IV (n=24; 1.6%)
Reduction in medication (n=2; 0.1%)		
Altering the oxygen intake in emergency care (n=1; 0.1%)		
Continuous positive airway pressure treatment combined with delaying a surgery (n=1; 0.1%)		
Deep brain stimulation (n=1; 0.1%)		
Exposure to theatrical smoke (n=1; 0.1%)		
Fecal microbiota transplant (n=1; 0.1%)		
**Non-invasive physical procedures (n=40; 2.6%)**		
Experimental interventions (n=13; 0.9%)	Medical device under investigation (n=27; 1.8%)	
Physiological home excercise (n=8; 0.5%)	Diagnostic device (n=18; 1.2%)	
Physiological group excercise (n=4; 0.3%)	Therapeutic device (n=9; 0.6%)	
Taking participants to different natural areas (n=1; 0.1%)		
**Non-physical procedures (n=12; 0.8%)**		
Experimental interventions (n=12; 0.8%)		
Psychological therapy in group (n=6; 0.4%)		
Intervention through computerized or mobile phone application (n=4; 0.3%)		
Psychological therapy given individually (n=2; 0.1%)		
