# Supplementary material for: Risk Assessment of Medical Study Procedures in the Documents Submitted to a Research Ethics Committee
Source: J Empir Res Hum Res Ethics. 2020 Feb 8;15(5):396–406. doi: 10.1177/1556264620903563 (PMC7604935; doi:10.1177/1556264620903563)
Supplement: SupplementaryTable2 – Supplemental material for Risk Assessment of Medical Study Procedures in the Documents Submitted to a Research Ethics Committee [file SupplementaryTable2.pdf]

Supplementary Table 2: Non-therapeutic procedures for data collection (n=1309; 87%)

Invasive physical procedures (n=287; 19%)		
Taking biological samples (n=199; 13%)	Imaging (n=49; 3.2%)	Measurements and examinations (n=39; 2.6%)
Blood sample (n=147; 9,7%)<br>Blood sample without extra needle penetration (n=22; 1.5%)<br>Tissue sample (n=19; 1.3%)<br>Biopsy, amniocentesis (n=4; 0.3%)<br>Lumbar puncture (n=4; 0.3%)<br>Spinal sample without extra needle penetration (n=2; 0.1%)<br>Setting a catheter into spinal canal (n=1; 0.1%)	X-ray imaging including dual-energy X-ray absorptiometry (n=22; 1.5%)<br>Computed tomography scan and/or positron emission tomography with imaging agent (n=13; 0.9%)<br>Computed tomography scan without imaging agent (n=5; 0.3%)<br>X-ray imaging with imaging agent (n=4; 0.3%)<br>Magnetic resonance imaging with imaging agent (n=2; 0.1%)<br>Angiography with imaging agent (n=1; 0.1%)<br>Optical coherence tomography with imaging agent (n=1; 0.1%)<br>Ultra-sound imaging with imaging agent (n=1; 0.1%)	Use of some diagnostic agent other than imaging agent* (n=26; 1.7%)<br>Cardiac stress test (n=7; 0.5%)<br>Physician examination using invasive methods (n=5; 0.3%)<br>Invasive allergy test (n=1; 0.3%)
**Non-invasive physical procedures (n=655; 43%)**		
Taking biological samples (n=128; 8.5%)	Imaging (n=63; 4.2%)	Measurements and examinations (n=464; 31%)
Urine sample (n=75; 5.0%)<br>Stool sample (13; 0.9%)<br>Tissue waste (12; 0.8%)<br>Exhalation air sample (n=8; 0.5%)<br>Swab sample (n=8; 0.5%)<br>Saliva sample (n=5; 0.3%)<br>Hair sample (n=3; 0.2%)<br>Breast milk sample (n=1; 0.1%)<br>Chewed food sample (n=1; 0.1%)<br>Nasal rinse sample (n=1; 0.1%)<br>Sperm sample (n=1; 0.1%)	Magnetic resonance imaging (n=33; 2.2%)<br>Ultra-sound imaging (n=23; 1.5%)<br>Infrared imaging (n=7; 0.5%)	Weighing and other body measurements (n=111; 7.4%)<br>Physician examination using non-invasive methods (n=94; 6.2%)<br>Monitoring basic body functions** (n=88; 5.8%)<br>Electrocardiography including long-term monitoring (n=53; 3.5%)<br>Neurological or neuropsychological tests (n=23; 1.5%)<br>Measuring physical performance in normal exercise (n=21; 1.4%)<br>Electroencephalography (n=18; 1.2%)<br>Electromyography on the skin (n=15; 1.0%)<br>Visual field testing and other measurements on eye (n=12; 0.8%)<br>Sleep polygraphy (n=4; 0.3%)<br>Spirometry (n=4; 0.3%)<br>Calorimetry (n=3; 0.2%)<br>Cardiotocography (n=3; 0.2%)<br>Measuring blood vessels on the skin (n=3; 0.2%)<br>Physical examination by a physiotherapist or a nurse (n=3; 0.2%)<br>Hearing tests (n=2; 0.1%)<br>Measuring moisture of skin and subcutaneous tissue (n=2; 0.1%)<br>Measuring sympathetic skin reactions (n=2; 0.1%)<br>Acoustic rhinometry (n=1; 0.1%)<br>Checking oral hygiene (n=1; 0.1%)<br>Pressure measurements on skin (n=1; 0.1%)
**Non-physical procedures (n=367; 24%)**		
Measurements and examinations (n=367; 24%)<br>Questionnaire (n=169; 11%)<br>Interview (n=114; 7.5%)<br>Diary (n=58; 3.8%)<br>Comparison between established treatments (n=11; 0.7%)<br>Observation (n=6; 0.4%)<br>Psychological testing (n=5; 0.3%)<br>Expression of feelings in writing (n=3; 0.2%)<br>Bringing a meal for analysis (n=1; 0.1%)		

*) Diagnostic agents such as oral glucose in glucose tolerance test or mydriatic agents in ophthamology

**) Measuring or monitoring blood pressure, heart rate, body temperature, or breathing rate
