# Supplementary material for: Risk Assessment of Medical Study Procedures in the Documents Submitted to a Research Ethics Committee
Source: J Empir Res Hum Res Ethics. 2020 Feb 8;15(5):396–406. doi: 10.1177/1556264620903563 (PMC7604935; doi:10.1177/1556264620903563)
Supplement: SupplementaryTable3 – Supplemental material for Risk Assessment of Medical Study Procedures in the Documents Submitted to a Research Ethics Committee [file SupplementaryTable3.pdf]

Supplementary Table 3: Study procedures (n=1510) classified according to the likely familiarity to an average participant

Likely to be familiar	Likely to be non-familiar
Questionnaire (n=169; 11%)	Study drug administration (n=73; 4.8%)
Blood sample (n=147; 9,7%)	Electrocardiography including long-term monitoring (n=53; 3.5%)
Interview (n=114; 7.5%)	Medical device under investigation (n=44; 2.9%)
Weighing and other body measurements (n=111; 7.4%)	Magnetic resonance imaging (n=33; 2.2%)
Physician examination using non-invasive methods (n=94; 6.2%)	Nutritional interventions (n=31; 2.1%)
Monitoring basic body functions** (n=88; 5.8%)	Use of some diagnostic agent other than imaging agent* (n=26; 1.7%)
Urine sample (n=75; 5.0%)	Neurological tests (n=23; 1.5%)
Diary (n=58; 3.8%)	Ultra-sound imaging (n=23; 1.5%)
Blood sample without extra needle penetration (n=22; 1.5%)	X-ray imaging including dual-energy X-ray absorptiometry (n=22; 1.5%)
Measuring physical performance in normal exercise (n=21; 1.4%)	Tissue sample (n=19; 1.3%)
Stool sample (13; 0.9%)	Electroencephalography (n=18; 1.2%)
Tissue waste (12; 0.8%)	Electromyography on the skin (n=15; 1.0%)
Comparison between established treatments (n=11; 0.7%)	Computed tomography scan and/or positron emission tomography with imaging agent (n=13; 0.9%)
Exhalation air sample (n=8; 0.5%)	Vision tests and other measurements on eye (n=12; 0.8%)
Swab sample (n=8; 0.5%)	Transcranial magnetic or electric stimulation (n=10; 0.7%)
Observation (n=6; 0.4%)	Physiological home excercise (n=8; 0.5%)
Saliva sample (n=5; 0.3%)	Surgical experiment (n=8; 0.5%)
Expression of feelings in writing (n=3; 0.2%)	Cardiac stress test (n=7; 0.5%)
Hair sample (n=3; 0.2%)	Infrared imaging (n=7; 0.5%)
Physical examination by a physiotherapist or a nurse (n=3; 0.2%)	Psychological therapy in group (n=6; 0.4%)
Hearing tests (n=2; 0.1%)	Computed tomography scan without imaging agent (n=5; 0.3%)
Breast milk sample (n=1; 0.1%)	Physician examination using invasive methods (n=5; 0.3%)
Bringing a meal for analysis (n=1; 0.1%)	Psychological testing (n=5; 0.3%)
Checking oral hygiene (n=1; 0.1%)	Biopsy, amniocentesis (n=4; 0.3%)
Chewed food sample (n=1; 0.1%)	Intervention through computerized or mobile phone application (n=4; 0.3%)
Nasal rinse sample (n=1; 0.1%)	Physiological group excercise (n=4; 0.3%)
Sperm sample (n=1; 0.1%)	X-ray imaging with imaging agent (n=4; 0.3%)
	Lumbar puncture (n=4; 0.3%)
	Sleep polygraphy (n=4; 0.3%)
	Spirometry (n=4; 0.3%)
	Calorimetry (n=3; 0.2%)
	Cardiotocography (n=3; 0.2%)
	Halotherapy (n=3; 0.2%)
	Measuring blood vessels on the skin (n=3; 0.2%)
	Magnetic resonance imaging with imaging agent (n=2; 0.1%)
	Measuring moisture of skin and subcutaneous tissue (n=2; 0.1%)
	Measuring sympathetic skin reactions (n=2; 0.1%)
	Psychological therapy given individually (n=2; 0.1%)
	Reduction in medication (n=2; 0.1%)
	Spinal sample without extra needle penetration (n=2; 0.1%)
	Acoustic rhinometry (n=1; 0.1%)
	Allergy test (n=1; 0.3%)
	Altering the oxygen intake in emergency care (n=1; 0.1%)
	Angiography with imaging agent (n=1; 0.1%)
	Continuous positive airway pressure treatment combined with delaying a surgery (n=1; 0.1%)
	Deep brain stimulation (n=1; 0.1%)
	Exposure to theatrical smoke (n=1; 0.1%)
	Fecal microbiota transplant (n=1; 0.1%)
	Optical coherence tomography with imaging agent (n=1; 0.1%)
	Pressure measurements on skin (n=1; 0.1%)
	Setting a catheter into spinal canal (n=1; 0.1%)
	Taking participants to different natural areas (n=1; 0.1%)
	Ultra-sound imaging with imaging agent (n=1; 0.1%)

*) Diagnostic agents such as oral glucose in glucose tolerance test or mydriatic agents in ophthamology

**) Measuring or monitoring blood pressure, heart rate, body temperature, or breathing rate
